# Impact of Diabetes Mellitus on Early Clinical Outcome and Stent Restenosis after Carotid Artery Stenting

**DOI:** 10.1155/2022/4196195

**Published:** 2022-07-11

**Authors:** Alexandru Achim, Dávid Lackó, Artúr Hüttl, Csaba Csobay-Novák, Ádám Csavajda, Péter Sótonyi, Béla Merkely, Balázs Nemes, Zoltán Ruzsa

**Affiliations:** ^1^Department of Internal Medicine, Division of Invasive Cardiology, University of Szeged, Szeged, Hungary; ^2^Department of Invasive Cardiology, Medicala 1 Clinic, University of Medicine and Pharmacy “Iuliu Hatieganu”, Cluj-Napoca, Romania; ^3^Semmelweis University, Cardiac and Vascular Center, Budapest, Hungary; ^4^Bács-Kiskun County Hospital, Teaching Hospital of the Szent-Györgyi Albert Medical University, Kecskemét, Hungary

## Abstract

**Background:**

Diabetes mellitus is closely related to both the severity of carotid disease and its outcome after revascularization. Carotid artery stenting (CAS) has emerged as a viable alternative to surgical endarterectomy but little is known about the impact of diabetes after CAS.

**Methods:**

A consecutive cohort of 1940 patients undergoing CAS in two institutions was divided into two groups, diabetics and nondiabetics, and major cerebrovascular events (MACCEs) were analyzed at 30 days post-CAS and at 1 year follow-up.

**Results:**

There were 730 patients with diabetes, with significantly higher BMI, hypertension, chronic dialysis, and dyslipidemia frequency (*p* < 0.05). There was no significant difference between the two groups in terms of early and late MACCEs (composite of transient ischemic attack, major stroke, myocardial infarction, and death), with an early rate of 3.5% nondiabetics vs. 5.3%, *p* = 0.08 and 2.4 nondiabetics vs. 2.3% diabetics, *p* = 0.1 at 12 months. Overall stroke/death rate in the asymptomatic patients was 2.4%, and the restenosis rate was higher in the diabetes population (2.3% vs. 1%, *p* = 0.04).

**Conclusion:**

The presence of diabetes was associated with an acceptable increased periprocedural risk for CAS, but no further additional risk emerged during longer term follow-up. Diabetes may precipitate the rate of early in-stent restenosis.

## 1. Introduction

Diabetes mellitus (DM) has been associated with an increased prevalence and severity of carotid artery disease [1], with patients with diabetes having three times the risk of coronary disease or stroke compared to individuals without this condition [2]. Moreover, when compared with nondiabetics, diabetics have a worse outcome after cardiovascular interventions [3–6]. Internal carotid artery stenosis accounts for 10–15% of all strokes [7]. Carotid endarterectomy lowers the long-term risk of stroke in patients with symptomatic carotid stenosis [8]. Diabetes is a major risk factor for stroke, and diabetics make up 11%–40% of patients receiving carotid endarterectomy (CEA) [7]. There is inconsistent evidence regarding the correlation of DM with outcomes after CEA and little data regarding carotid artery stenting (CAS) [9]. CAS has emerged as a reliable alternative to endarterectomy because in randomized controlled trials (RCTs) comparing CAS with CEA for symptomatic carotid stenosis, stenting was associated with a higher risk of procedure-related stroke, particularly in elderly patients, but with lower risks of myocardial infarction, cranial nerve palsy, and access site hematoma [7, 10–12]. A closer look showed that the increase in procedure-related risk was powered by nondisabling stroke, with no evidence for a difference in rates of major or disabling stroke or mortality between the treatments [13, 14]. The impact of diabetes on the outcome of patients undergoing CAS remains unknown and because this procedure is expanding in both, prevalence and complexity, a rigorous examination of its prognosis remains imperative. This prospective, multicenter, double-cohort, observational study, based on a large sample size, aimed to compare the effectiveness of percutaneous carotid revascularization in diabetic vs. nondiabetic patients and to evaluate the impact of DM on the outcomes of CAS.

## 2. Methods

### 2.1. Study Design and Patients

This retrospective two-center study was conducted on patients percutaneously treated for carotid artery stenosis during a 12-year period (January 2009-July 2021) at two high-volume Hungarian referral centers, Semmelweis University Heart and Vascular Center from Budapest and Bács-Kiskun County Teaching Hospital from Kecskemét. During this period, 1940 patients were treated due to either >50% symptomatic or >70% asymptomatic carotid stenosis, from which 730 had diabetes mellitus (37.6%) and 1210 patients were nondiabetics (62.3%). All data concerning these patients were prospectively collected in a dedicated database which contained preoperative and intraoperative data as well as perioperative results in terms of mortality and neurological and cardiac morbidity. Our Institutional Review Committee approved the study, and all patients provided written informed consent prior to study inclusion.

Inclusion criteria were as follows: (1) asymptomatic patients with carotid stenosis ≥ 70% and (2) symptomatic patients with carotid stenosis ≥ 50%, as detected by duplex ultrasound examination and confirmed by computer tomography angiography or magnetic resonance angiography using the North American Symptomatic Carotid Endarterectomy Trial (NASCET) criteria [15]. The strategy for the revascularization method (i.e., CAS or CEA) was based on the current guideline recommendations e.g., ESVS (European Society Vascular Surgery), ACC/AHA (American College of Cardiology and American Heart Association), clinical judgment, and the desire of the patient [12, 16]. The following features excluded CAS: (1) history of acute or recent stroke (<2 weeks), (2) extreme deformity of the aortic arch or extremely tortuous carotid anatomy, or extreme calcification, (3) visible thrombus, and (4) known allergies to aspirin, clopidogrel, or contrast media.

As a further step, patients entered into the database were divided into two groups and analyzed. The first group included patients without diabetes while the second group included patients with diabetes. Patients with diabetes were considered as all those patients who had previously been diagnosed with diabetes mellitus (oral or insulin controlled glycaemia). Baseline demographic and clinical characteristics, interventional devices, stent type, procedural outcomes, and clinical complications were recorded.

Patient risk was also evaluated. Patients with a high risk of CEA were defined as those who met at least one of the following criteria: congestive heart failure (NYHA class III/IV), recent myocardial infarction (MI) in the last 4 weeks, severe angina (Canadian Cardiovascular Society class III/IV), multivessel coronary artery disease, severe COPD (GOLD III/IV), contralateral internal carotid artery occlusion; unstable carotid lesion, restenosis after CEA, unfavorable anatomy, and age ≥ 80 years.

### 2.2. CAS Procedures

Dual antiplatelet therapy was administered within 24 hours before the procedure. Intraoperative anticoagulation was achieved using 100 units/kg heparin. CAS was performed under local anesthesia without sedation. All aortic arch types were included. Majority of the cases were carried out via radial access, using Judkins-Right 3.5-4.0, 6.5, or 7.5-French sheathless guiding catheters (Asahi Intecc, Aichi, Japan) and in case of femoral access, the 7-French Guider-Softip XF (Boston Scientific, Marlborough, MA, USA) was preferred. CAS was performed according to the standard clinical practice, in the majority of cases using the Carotid WALLSTENT (Boston Scientific Corporation, Natick, MA, USA), Cristallo Ideale (Medtronic-Invatec, Frauenfeld, Switzerland), Roadsaver stent (Terumo, Tokyo, Japan), and Precise (Cordis Corporation, Bridgewater, NJ, USA) stents. In all procedures, we used either the EZ Filter wire (Boston Scientific, Marlborough, MA, USA) or Emboshield (Abbott Vascular, Santa Clara, CA, USA) cerebral protection device. Postdilation, to the diameter of the internal carotid artery ICA, was highly recommended (a more detailed procedural data is being presented in Table 1). Completion angiography was then performed, and a closure device was used to achieve hemostasis in all femoral cases. A successful angioplasty was defined as no more than 30 percent postintervention stenosis by the NASCET criteria.

### 2.3. Outcomes of Interest and Follow-Up

The primary outcome was the combined risk of any stroke, MI, or death within 30 days (perioperative). Secondary end points were the rate of stroke, death, and restenosis 1 year after the procedure. The relationship between the restenosis rate and other relevant factors such as stent design, postdilatation, antiplatelet, and/or statin therapy was also analyzed.

A major cerebrovascular clinical event (MACCE) was defined as any stroke, MI, or death. Any death, stroke, or MI < 30 days from the procedure was considered procedure-related. Stroke was defined as focal neurologic function acute disturbance that lasted over 24 h and resulted from intracranial vascular disturbance. The definition of minor strokes was neurologic deficits that resolved completely within 30 days or led to no functional impairment in daily activities. All other strokes were considered major strokes. MI was defined as the appearance of new pathologic *Q* waves on a standard electrocardiogram in two or more contiguous leads and/or a total creatinine kinase rise greater than twice the upper limit of normal with an elevated creatinine kinase myocardial band fraction. The short-term follow-up data were obtained through clinical visit or telephone. Patients were divided into the MACCE (+) group and the MACCE (–) group. Carotid restenosis was set at >50%, quantified by duplex ultrasound.

### 2.4. Statistical Analysis

Continuous variables with normal distribution are demonstrated as mean ± standard deviation, while categorical variables are demonstrated as number and percentage. The differences in categorical variables between the diabetic group and nondiabetic group were analyzed by chi-squared test or Fisher exact test. The differences in continuous variables were analyzed by *t*-test. Odds ratios (ORs) and 95% confidence intervals (CIs) were calculated for 30-day postoperative MACCEs. SPSS version 19.0 (IBM, Chicago, IL, USA) was used for the data analysis. A *p* < 0.05 was considered statistically significant.

## 3. Results

In our study, 1940 patients were enrolled during the recruitment period, out of which 48 were lost to follow-up before 1 year.

Patients' characteristics at baseline were similar in the two groups (see Table 1). A total of 26.3% of patients were symptomatic. CAS was performed in symptomatic patients following ischemic stroke in 52 cases, TIA in 34 cases, and amaurosis fugax in 30 cases; 38.8% of them were in the high-risk group for CEA, and the remaining 61.2% were in the normal-risk group. There were no differences between these two groups in the terms of age (*p* = 0.8) and gender (*p* = 0.2), but the patients with diabetes had significantly higher BMI (*p* < 0.002). The frequency of hypertension (*p* < 0.0001), dyslipidemia (*p* < 0.002), and family history of cardiovascular disease (*p* < 0.03) was found to be significantly higher in the diabetic cohort. There was no difference found among these two groups in terms of coronary disease (*p* = 0.2) and smoking (*p* = 0.2). As expected, the history of previous carotid percutaneous transluminal angioplasty (PTA) was more prevalent in the diabetic group (*p* < 0.0004). Most of the patients with diabetes were on statin therapy (*n* = 632, *p* < 0.01) and had minimum an antiplatelet agent in their therapy (*n* = 693, *p* < 0.01).

### 3.1. Procedural Data

There were 1323 (68%) asymptomatic, 501 (25%) symptomatic carotid stenoses, and 116 (6%) acute carotid syndromes. Radial access was the most frequently used (*n* = 1400, 72%). The stent mostly used was Carotid WALLSTENT (Boston Scientific Corporation, Natick, MA, USA) (*n* = 1284, 66%), followed by Roadsaver stent (Terumo, Tokyo, Japan) (*n* = 214, 11%). Mean procedural duration was 35.1 ± 10.9 minutes, from which mean fluoroscopy time was 9.10 ± 6.9 minutes, generating an average radiation dose of 390 ± 32.2 mGy. The average dose of contrast administered was 109 ± 15 ml of iodinated agent. No difference in hospitalization duration was observed (nondiabetics 5 ± 2 vs. diabetics 5 ± 3 days).

### 3.2. Complications and Follow-Up (See Table 2)

Minor procedural complications, such as bradycardia (*n* = 39, 2%) or asystole (*n* = 3, 0.1%), were more than double in the diabetic population (1.5% nondiabetics vs. 3.3% diabetics). Early (< 30 days) results showed 81 (4.1%) major cerebrovascular events. There was no statistically significant difference between the two groups (3.5% nondiabetics vs. 5.3%, *p* = 0.08). Rates of MI, transient ischemic attack (TIA), and cranial nerve injuries were also evenly distributed. A separate subanalysis showed a 2.4% stroke/death rate in the asymptomatic patients, with no difference between the two groups (2.38% nondiabetics vs. 2.43% diabetics, *p* = 0.1). Follow-up was at 12 months; during this period, 41 patients deaths (1.4%) and 85 (4.3%) ischemic strokes were reported. Overall, 163 additional MACCEs (8.7%) were recorded at 1-year follow-up, with no difference between the diabetic and nondiabetic population, 2.4% nondiabetics vs. 2.3% diabetics, *p* = 0.1 (Table 2).

At 1-year, patients with diabetes had a significantly higher restenosis rate comparing to nondiabetics (2.3% vs. 1%, *p* = 0.04). Further analysis (inverse probability treatment weighting) showed no difference between the two groups in terms of stent design (restenosis rate for WALLSTENT 54% vs. Roadsaver stent 46%, *p* = 0.1). The rate of postdilatation was significantly lower in the restenosis patients (71% vs. 86%, *p* = 0.04), with similar distribution across the diabetics and nondiabetics (diabetics 47% vs. nondiabetics 53%, *p* = 0.8).

## 4. Discussion

Our study showed that patients with diabetes and severe carotid stenosis share similar periprocedural stroke and death risks of nondiabetic patients when carotid stenting is applied for treatment (perioperative stroke and death rate: nondiabetics 1.7% vs. diabetics 2.0%; *p* = 0.08). Nevertheless, there is weak evidence towards a worse perioperative early outcome for patients with diabetes. Yet, according to our study, at one year follow-up, the rate of major cerebrovascular events is leveling, with similar outcomes between the two groups. Our findings are consistent with the conclusion of other previously published data [17–20]. Moreover, our early <30 days overall stroke/death rates fell under the 3% threshold in elective cases recommended by the American and European societies [12, 21]. The results of our study are of even greater relevance in the context in which the rates of 30-day stroke/death after CEA in asymptomatic patients with insulin-dependent DM exceeded international vascular societies' guideline thresholds for acceptable outcomes in asymptomatic patients, especially those with anatomic high-risk criteria [9]. General metabolic syndrome was also a risk for short-term MACCEs after CEA, but not CAS, in a 2000-chinese cohort reported by Jiao et al. [22]. However, it must be admitted that these data come from a retrospective, observational study, and our analysis did not differentiate between insulin-dependent diabetics and noninsulin-dependent diabetics.

It should be noted that the rate of early restenosis in the diabetic population was double (2.3% vs. 1%, *p* = 0.04). An important limitation of the present study is that it does not provide follow-up longer than 1 year, because diabetes could further increase risk for restenosis over time. However, this hypothesis comes from studies published more than 10 years ago, and it must be acknowledged that in the meantime, progress has been made in terms of endovascular treatment tools and new antiplatelet agents. Only Casana's study was published in 2018, showing the same trend, increased early periprocedural risk, but no further additional risk during longer term follow-up in the diabetic population undergoing CAS [23]. Restenosis rates reported by Casana et al. were also significantly higher among patients with diabetes (21.2% diabetes vs. 12.5% no diabetes at 36-months follow-up). Stent restenosis is presumed to be the result of neointimal hyperplasia, and this can be accelerated by diabetes [24], especially if the initial glycemic state, mirrored by HbA1c, is high during CAS. The early phase of stent healing seems to be influenced by the poor glycemic state rather than the diabetic condition, with good glycemic control, [25]; so, it is understandable that aiming for strict hyperglycemic optimization prior to the procedure is important. Another limitation of our study is that HbA1c was not followed; so, an in-depth analysis of the restenosis rate in association with the baseline glycemic status could not be made. Similar mechanisms that demonstrate accelerated restenosis in diabetics have been described in other interventional fields [26–28]. It is assumed that the stenting of the coronary atherosclerotic plaque is different from the coronary plaque by the fact that in the case of CAS, the plaque is only pushed outwards, not cracked, and modified to the media, which would later stimulate intima proliferation [26].

In the literature, anatomical and technical risk factors for restenosis include the number of stents deployed, the presence of large and calcified plaques, and the existence of residual stenosis after the procedure or even the stent design used, to the detriment of stiffer stents with small cell sizes, but moderate radial force, such as WALLSTENT (Boston Scientific Corporation, Natick, MA, USA) [29, 30]. Dyslipidemia, statin therapy, female sex, and smoking were associated with CAS restenosis as well [31, 32]. Most likely, in our case, all types of stents performed well, due to the high postdilatation rate, avoiding residual lesions and metal recoil. A possible result bias could be the homogeneous population who received CAS in only 2 centers; on the other hand, the uniform skill experience of a few operators who have followed a fixed procedural protocol might have positively influenced the outcome of these patients. As diabetes appears to be an independent predictor of restenosis in several studies already, an optimal result in this subpopulation should be achieved, especially since, in the present study, the lack of postdilatation was correlated with repeat target lesion revascularization.

CAS has developed rapidly in recent years and has gradually become an alternative treatment for CEA [33, 34]. An increasing number of hospitals can carry out stent implantation, and an increasing number of patients receive stenting because it is a minimally invasive and efficient treatment; using radial access is also possible in CAS [34, 35]. Several large RCTs and meta-analyses have compared the efficacy and safety of CEA with CAS. However, to date, no RCTs have directly compared the effects of diabetes on the perioperative and long-term outcomes of patients with carotid artery stenosis after CEA or CAS surgery. Only a few observational studies have analyzed the effects of diabetes on CAS. Therefore, there is no consensus on which type of carotid revascularization should be performed in patients with DM. Our main findings provide evidence that CAS in diabetics can be performed under the same safety and feasibility conditions as CAS in general, with an emphasis on optimal control of this metabolic disease. A longer follow-up (minimum 5 years) is of great importance to validate this statement.

There are several limitations of our study that are worthy of mentioning. First, the study was retrospective, nonrandomized; although, patients were enrolled consecutively, receiving the same type of treatment, according to an internal procedural protocol. Second, the follow-up of patients is limited to 1 year; a definitive answer on clinical hard-endpoints will have to be provided at 5 and 10 years. Third, the severity of diabetes and the type of antidiabetic therapy were not known, and this could have an independent impact on MACCE.

## 5. Conclusion

The current study suggested that the presence of diabetes was associated with an acceptable increased periprocedural risk for CAS, but no further additional risk emerged during longer term follow-up. Diabetes may precipitate the rate of early in-stent restenosis.

## Figures and Tables

**Table 1:** Baseline characteristics in 1940 patients. PCI: percutaneous coronary intervention; CABG: coronary artery bypass grafting; PTA: percutaneous transluminal angioplasty; COPD: chronic obstructive pulmonary disease.

	Nondiabetics	Diabetics	*p* value
	(*n* = 1210)	(*n* = 730)	
Age (years)	68.7 ± 12	67.4 ± 11	0.14
Male sex	547 (45%)	353 (48%)	0.17
Vascular risk factors			
Hypertension	42 (34%)	283 (39%)	0.03
Dyslipidemia	211 (17%)	136 (19%)	0.50
Chronic dialysis	114 (9%)	78 (10%)	0.36
Smoking	281 (23%)	159 (22%)	0.46
Family history	271 (22%)	166 (23%)	0.49
Previous PCI	289 (24%)	198 (27%)	0.11
Previous CABG	101 (8%)	79 (11%)	0.06
Previous carotid PTA	75 (6%)	59 (8%)	0.11
Peripheral artery disease	189 (15%)	134 (18%)	0.11
Atrial fibrillation	233 (19%)	134 (18%)	0.40
COPD	155 (12%)	89 (12%)	0.69
Degree of symptomatic carotid stenosis			
50–69%	44 (15%)	31 (16%)	0.96
70–99%	251 (85%)	175 (84%)	0.96
Indication for stenting			
Asymptomatic carotid stenosis	843 (69%)	480 (65%)	0.06
Symptomatic carotid stenosis	295 (24%)	206 (28%)	0.06
Acute carotid syndrome	72 (7%)	44 (7%)	0.94
Procedural data			
Radial access	883 (72%)	517 (70%)	0.30
Femoral access	281 (23%)	188 (25%)	0.20
Aortic arch type II/III	553 (45%)	312 (44%)	0.20
Postdilatation	1009 (83%)	602 (84%)	0.62
Predilatation	321 (26%)	201 (28%)	0.62
Closed-cell stent (WALLSTENT®)	784 (55%)	499 (54%)	0.10
Mesh-stent (Roadsaver ®)	129 (11%)	85 (12%)	0.50
Length-of-stay, days^a^	5 (3-8)	5 (3-9)	0.43

^a^Continuous variables are summarized using medians and interquartile ranges (IQR).

**Table 2:** Perioperative (< 30 days) and 1-year follow-up results among diabetic and nondiabetic patients. TIA: transient ischemic attack; MI: myocardial infarction.

	Nondiabetics	Diabetics	*p* value	Nondiabetics	Diabetics	*p* value
	(*n* = 1210)	(*n* = 730)		(*n* = 1182)	(*n* = 690)	
	At 30 days			At 12 months		
Minor TIA/stroke	27 (2.3%)	21 (2.8%)	0.37	33 (2.7%)	19 (2.7%)	0.96
Major stroke	14 (1.1%)	10 (1.3%)	0.68	20 (1.7%)	13 (1.8%)	0.76
MI	5 (0.4%)	5 (0.6%)	0.41	21 (1.7%)	16 (2.3%)	0.41
Death	2 (0.1%)	1 (0.1%)	0.88	25 (2.1%)	16 (2.3%)	0.77
Restenosis	0 (0%)	0 (0%)		12 (1%)	16 (2.3%)	0.04

## Data Availability

Data is available upon request, and it is not deposited in a public repository due to patient personal data protection. Achim Alexandru can be contacted at dr.alex.achim@gmail.com or +40264597852 for this matter.

## Abbreviations

DM: Diabetes mellitus
CEA: Carotid endarterectomy
CAS: Carotid artery stenting
RCTs: Randomized controlled trials
MI: Myocardial infarction
MACCE: Major cerebrovascular clinical event
TIA: Transient ischemic attack.
